# The Impact of HIV on B Cell Compartment and Its Implications for COVID-19 Vaccinations in People with HIV

**DOI:** 10.3390/vaccines12121372

**Published:** 2024-12-05

**Authors:** Lixing Wang, Branka Vulesevic, MariaLuisa Vigano, Alia As’sadiq, Kristina Kang, Cristina Fernandez, Suzanne Samarani, Aslam H. Anis, Ali Ahmad, Cecilia T. Costiniuk

**Affiliations:** 1 Department of Microbiology and Immunology, McGill University, Montreal, QC H3A 2B4, Canada; lixing.wang@mail.mcgill.ca (L.W.); cristina.fernandez@mail.mcgill.ca (C.F.); 2 Infectious Diseases and Immunity in Global Health Program, Research Institute of the McGill University Health Centre, Montreal, QC H4A 3J1, Canadamarialuisa.vigano@mail.mcgill.ca (M.V.); alia.assadiq@mail.mcgill.ca (A.A.); kristina.k.kang@mail.mcgill.ca (K.K.); suzanne.samarani@muhc.mcgill.ca (S.S.); 3 Division of Experimental Medicine, Faculty of Medicine and Health Sciences, McGill University, Montreal, QC H3A 0G4, Canada; 4 Centre for Advancing Health Outcomes Centre for Health Evaluation and Outcome Sciences, St. Paul’s Hospital, Vancouver, BC V6Z 1Y6, Canada; aslam.anis@ubc.ca; 5 Centre de Recherche, Hôpital Ste Justine, Montréal, QC H3T 1C5, Canada; ali.ahmad.hsj@ssss.gouv.qc.ca; 6 Département de Microbiologie, Infectiologie et Immunologie, Université de Montréal, Montréal, QC H3T 1C5, Canada; 7 Division of Infectious Diseases and Chronic Viral Illnesses Service, McGill University Health Centre, Montreal QC H4A 3J1, Canada

**Keywords:** anti-SARS-CoV-2 vaccines, ART, B cells, COVID-19, HIV, PWH, vaccinations

## Abstract

HIV causes intense polyclonal activation of B cells, resulting in increased numbers of spontaneously antibody-secreting cells in the circulation and hypergammaglobulinemia. It is accompanied by significant perturbations in various B cell subsets, such as increased frequencies of immature/transitional B cells, activated memory B cells, atypical memory B cells, short-lived plasmablasts and regulatory B cells, as well as by decreased frequencies of resting memory and resting naïve B cells. Furthermore, both memory and antigen-inexperienced naïve B cells show exhausted and immune-senescent phenotypes. HIV also drives the expansion and functional impairment of CD4^+^ T follicular helper cells, which provide help to B cells, crucial for the generation of germinal center reactions and production of long-lived plasma and memory B cells. By suppressing viral replication, anti-retroviral therapy reverses the virus-induced perturbations and functional defects, albeit inadequately. Due to HIV’s lingering impact on B cells, immune senescence and residual chronic inflammation, people with HIV (PWH), especially immune non-responders, are immunocompromised and mount suboptimal antibody responses to vaccination for SARS-CoV-2. Here, we review how functionally and phenotypically distinct B cell subsets are induced in response to a vaccine and an infection and how HIV infection and anti-retroviral therapy (ART) impact them. We also review the role played by HIV-induced defects and perturbations in B cells in the induction of humoral immune responses to currently used anti-SARS-CoV-2 vaccines in PWH on ART. We also outline different strategies that could potentially enhance the vaccine-induced antibody responses in PWH. The review will provide guidance and impetus for further research to improve the immunogenicity of these vaccines in this human population.

## 1. Introduction

By the end of 2023, there were more than 39 million people living with HIV (PWH) in the world (https://www.who.int/news-room/fact-sheets/detail/hiv-aids; accessed on 24 June 2024). Current antiretroviral therapies (ART; please see a list of abbreviations after conclusions) effectively suppress viral replication to undetectable levels. However, despite effective suppression of HIV replication, ART does not normalize CD4^+^ T cell counts in about 10–40% of PWH [1]. Such individuals, called immune non-responders (INRs), suffer from higher rates of comorbidity and mortality from AIDS-defining and non-AIDS-defining events. In addition, ART does not eliminate the virus from the body and does not provide a cure for the infection. For this reason, PWH are obliged to take antivirals for life, as viral replication rebounds within days to weeks following discontinuation of ART, re-initiating its pathological process. ART itself also induces significant metabolic toxicities in both immune and non-immune cells, adversely affects immune cell proliferation and skews their cytokine production from a polyfunctional response towards a TNF-α-dominated stress response [2]. Consequently, PWH experience accelerated aging and immune senescence, as well as metabolic, cardiovascular and cognitive abnormalities. They are at higher risk for cardiovascular diseases, atherosclerosis, obesity, diabetes and cancers [3,4]. Furthermore, they are also more susceptible to many vaccine-preventable infections such as SARS-CoV-2, influenza, measles, pneumococcal infections and Mpox [5]. The increased risk for the vaccine-preventable diseases in PWH stems, in part, from suboptimal immune responses to the vaccines. The suboptimal responses are observed more frequently in INR who have their CD4^+^ T cell counts below 350 per mm^3^, despite a suppressed viral load [6]. In these individuals, due to lingering effects of HIV infection, B cells are not able to generate vaccine-specific antibodies of sufficient titers, affinity and durability to provide protection from the infections [7]. Here, we discuss HIV-induced perturbations at the cellular and molecular levels in B cell compartment that persist in PWH despite ART and how they affect antibody responses to anti-COVID-19 vaccines. The diminished help for B cells from CD4^+^ T cells will also be discussed. Finally, we will highlight outstanding questions that need to be addressed for improving the immunogenicity of vaccines in PWH.

## 2. Induction of Antibody-Secreting Plasma Cells and Memory B Cells

B cells constitute an essential component of the adaptive arm of the immune system. Their main function is to secrete antigen-specific antibodies to neutralize invading pathogens, although they can perform several other functions, such as antigen presentation to T cells and immune regulation through the secretion of various cytokines and chemokines. Under certain pathological conditions, they may also secrete antibodies to self-antigens and cause autoimmune diseases such as rheumatoid arthritis (RA), systemic lupus erythematosus (SLE), myelin oligodendrocyte glycoprotein antibody-associated disease (MOGAD) and Neuromyelitis Optica (NMO). B cells were named so since they were first discovered in the bursa of Fabricius in chickens in 1960 [8]. The initial stages of the development of B cells from common lymphoid precursors until the immature/transitional (IT) stage occurs in the bone marrow. During this development, B cells acquire their B cell receptors (BCRs) through VDJ recombination events and undergo the deletion of autoreactive B cell clones as well [9]. The short-lived IT B cells exit bone marrow through blood and localize in secondary lymphoid organs (SLOs), where they develop into mature naïve B cells [10,11]. 

**Figure 1:**
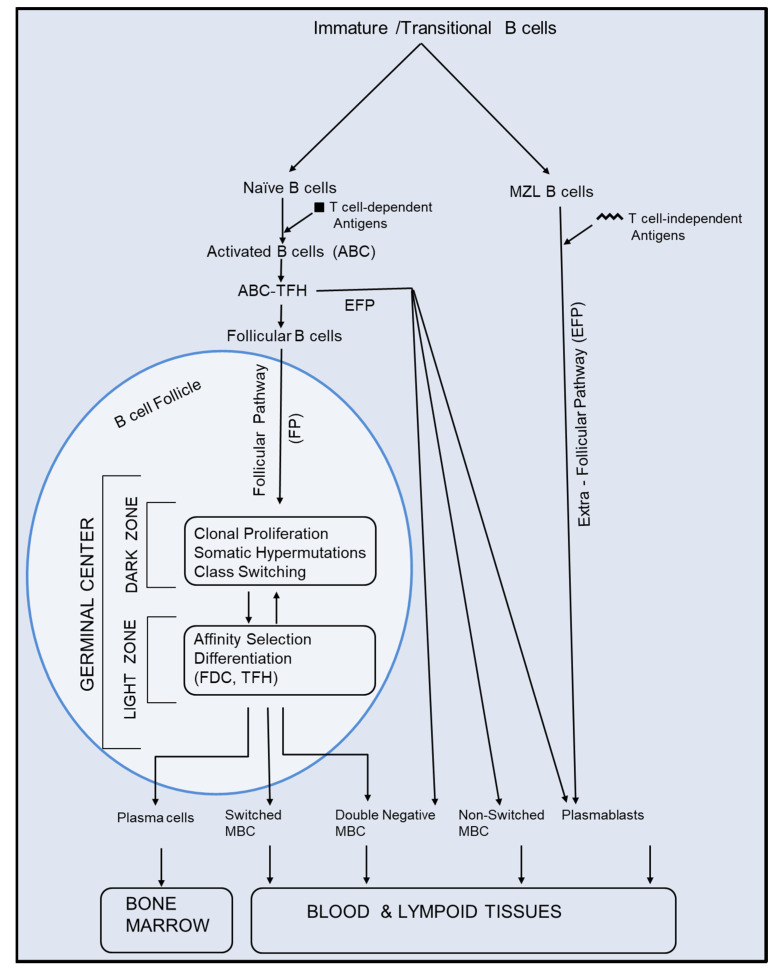
**Differentiation and maturation of B cells into antibody-secreting cells and memory B cells**. The figure shows two pathways, extrafollicular and follicular, for the differentiation and maturation of B cells into antibody-secreting cells and memory B cells. EFP: Extrafollicular pathway; FDCs: Follicular dendritic cells; Non-switched memory B cells; TFHs: T follicular helper cells (see text for details).

Naïve B cells recirculate in the blood and lymphoid tissues. After interaction with their specific antigens, naïve B cells become activated, upregulate the C-C chemokine receptor (CCR7) and the Epstein Barr virus-induced gene-2 (EBI2) and migrate to the border between T and B cell zones in the regional lymph nodes [12]. Here, activated B cells (ABCs) interact with cognate T follicular helper (TFH) cells (Figure 1), undergo clonal proliferation and follow one of the three fates, i.e., may differentiate into (1) terminally differentiated antibody secreting plasmablasts (PB), (2) non-switched (NS) memory B cells (MBCs) or (3) follicular B cells (FBCs) that form germinal centers (GCs) in B cell follicles (Figure 1). These fates are dependent upon the B cell’s access to, and help from, their cognate TFH cells, as well their BCR’s affinity for the antigen. The cells that receive little or no help from the TFH differentiate into PB and non-switched memory B cells (NSMBC) through the GC-independent extrafollicular pathway (EFP). PB are short-lived and survive only for 3–5 days. Both PB and NSMBC (or Pre-GC MBC) have undergone limited somatic hypermutations (SHMs; point mutations in their immunoglobulin variable/IgV genes) and class switching [13,14]. They develop rapidly and provide protection from the infection before high-affinity antibodies against the pathogen via GC reactions are produced. The early pre-GC MBC have low-affinity BCR; however, upon exposure with a mutant viral strain from the previous infection, they undergo further SHM and affinity selection in secondary GC and protect the host from viral variants. Follicular B cells (FBCs) interact with and receive help from TFH, undergo extensive clonal proliferation, form the dark zone (DZ) of the GC, in which they undergo, SHM and class switching. The TFH-secreted cytokine IL-21 induces expression of the activation-induced deaminase (AID) in B cells that mediates SHM and class switching in B cells. After undergoing SHM and class switching, clonally expanded B cells exit the cell cycle and migrate to the light zone (LZ) of GC, where they interact with follicular dendritic cells (FDCs) and TFH (Figure 1). Here, B cells are selected for the affinity of their BCR for its specific antigen. FDC capture antigens opsonized by complement and antibodies. Depending upon the affinity of their BCR for their specific antigens, B cells may develop into MBC (predominantly as SMBC) or antibody-secreting plasma cells (PCs). B cells with lower affinity and survival advantage (for example, higher expression of Bcl-2) exit the LZ of the GC as MBC, whereas those with a relatively higher affinity for antigens exit the LZ as PC.

TFH are a specialized subset of CD4^+^ T cells; they provide help to B cells and play a critical role in B cell differentiation through the follicular pathway (FP). Upon consecutive interactions with dendritic cells (DCs) and B cells, CD4^+^ T cells differentiate into pre-GC TFH in the T cell zone at the periphery of B cell follicles in secondary lymphoid organs (SLOs). Among others, IL-6 produced from these cell–cell interactions plays an important role in TFH differentiation; it induces B cell lymphoma-6 (BCL6), which is an essential transcription factor for TFH differentiation [15]. 

MBC recirculate in blood and reside in lymphoid and peripheral tissues, whereas PC migrate predominantly to, and reside in, bone marrow. Those FBC with intermediate affinity of their BCR with the antigen re-enter the DZ for further clonal expansion, SHM and class switching. B cells that cannot productively assemble their BCR after SHM undergo apoptosis. In the LZ, B cell–TFH interaction also leads to the generation of memory TFH (mTFH), a small percentage of which are found in the blood as circulating TFH (cTFH) [13]. In the bone marrow, PCs may live for a few months, decades or for life as long-lived plasma cells (LLPCs). They may also reside in other tissues such as the spleen and lamina propria of mucosae and develop unique transcriptional programs.

**Figure 2:**
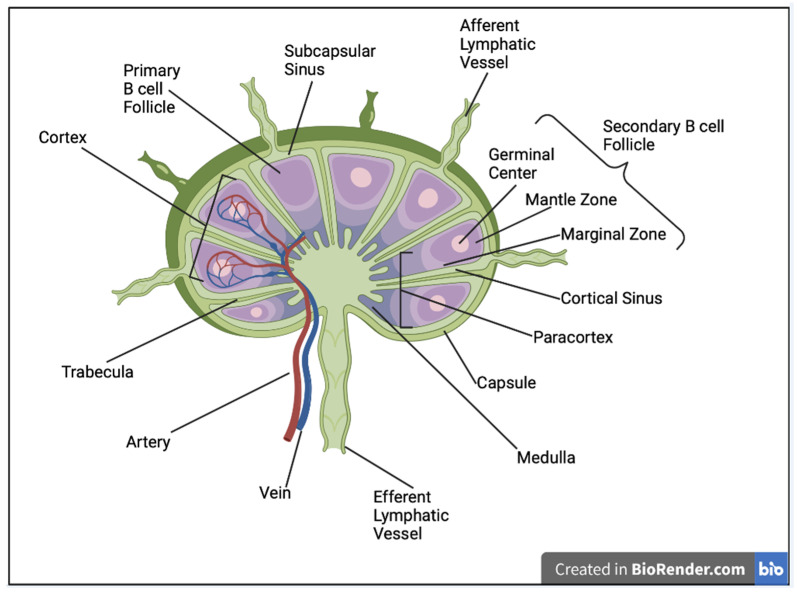
**Schematic depiction of lymph node anatomy.** The figure shows the location of a secondary B cell follicle containing a germinal center, mantle zone and marginal zone in relation to other structures in a typical lymph node. Blood is filtered in the marginal zone and is scavenged by MZ-like B cells and macrophages for blood-borne pathogens. MZL B cells and macrophages are in direct contact with blood through fenestrated sinusoidal endothelium. The cortex mainly contains mainly B cells, the paracortex is enriched in T cells and dendritic cells and the medulla has plasma cells and macrophages. Modified from Reference [66].

Plasma cells constitutively produce high-affinity antibodies and protect the host from reinfection with a homologous virus. These antibodies can be detected and measured in the circulation. MBC, compared to LLPC, have a broader repertoire of BCR diversities and are meant to protect the host from the same (previously encountered) and mutated antigens in secondary GC. Therefore, they are of high importance in preventing infection with viral variants. They may have long lives, recirculate in the blood, reside in lymphoid tissues and are equipped to rapidly develop and differentiate into antibody-secreting cells (ASCs) upon subsequent infection or booster vaccine doses. A subset of MBC is known to reside in lymph nodes, making local immunizations more effective [13]. Upon encountering the antigen, CD169^+^ macrophages in the subscapular sinus of a lymph node (Figure 2) present antigens to, and activate MBC; activated MBC interact with mTFH and differentiate into plasma cells. They may also enter into GC to undergo further affinity maturation and selection for mutated antigens. Thus, as far as B cells are concerned, the aim of vaccination is to induce antigen-specific long-lived memory B cells, as well as LLPC, to provide protection from the pathogen and its mutant forms in the future [12,13].

At each stage in the process, differentiation from naïve B cells into MBC or antibody-secreting cells (ASCs), B cells express specific molecules/markers on their surface or intracellularly. The surface-expressed markers are recognized by a cluster of differentiation (CD) numbers, such as CD19, CD20, CD21 and so on, into different subsets. Describing the functions of these molecules is beyond the scope of this review, and readers are referred to relevant reviews [16,17]. The molecules play important roles in modulating the development, differentiation and functions of B cells. Determining absolute numbers and/or percentages of these subsets in the blood informs about the qualitative and quantitative aspects of the humoral immune response induced by a vaccine or an infection. The subsets can be enumerated by flow cytometry or mass spectrometry using marker-specific monoclonal antibodies. Their investigations in PWH have provided a wealth of literature about the virus-induced defects and perturbations in the B cell compartment.

## 3. HIV-Induced Defects and Perturbations in the B Cell Compartment

HIV is known to cause significant perturbations and defects in all major immune cells, including B cells in the body. Although HIV does not directly infect B cells, viral proteins do interact with B cells. For example, HIV envelope protein gp-120 acts as a super-antigen for B cells. It interacts with and stimulates B cells expressing most, if not all, VH-3 family genes (VH3) [18,19]. The initial stimulation leads to B cell proliferation, secretion of HIV non-specific antibodies and a gradual depletion of the VH3-expressing B cells as the disease progresses to AIDS in untreated HIV-infected individuals. Gp-120 also binds the integrin α4β7, which is expressed on several types of immune cells, including CD4^+^ T, NK and B cells, and directs their homing to the gut and other mucosal tissues. Through this integrin, gp-120 induces expression of the inhibitory receptor FcRL4 (the receptor for Fc region of IgA) on B cells, as well as secretion of TGF-β [20]. The negative factor (Nef), a viral protein expressed and secreted by HIV-1-infected cells, induces B cell proliferation and inhibits immunoglobulin (Ig) class switching from IgM to other Ig classes. The class switching is crucial for effective antiviral immunity. It depends on B cell activation by T cells through the CD40 ligand and cytokines (such as IL-21). This activation process is regulated by negative feedback mechanisms involving proteins such as IκΒα and suppressors of cytokine signaling (SOCS) proteins. Nef infiltrates B cells when they interact with macrophages in which Nef forms actin-propelled conduits. Nef is transferred to B cells from macrophages through these conduits. Consequently, HIV can suppress antiviral IgG and IgA responses in virus-infected individuals. These responses are maintained in the individuals infected with Nef mutant HIV [21,22].

HIV induces two main defects in B cells: activation and exhaustion, activation leading to exhaustion [16,23]. B cells in HIV-infected individuals often show a loss/decreased expression of CD21 (also known as type II complement receptor) and an upregulation of CD95. The loss of CD21 is a sign of B cell exhaustion and anergy and can occur in the absence of viral replication, whereas CD95 (Fas) upregulation results from ongoing viral replication and immune activation [24]. CD21 enables B cells to capture complement-opsonised antigens, amplifies BCR-induced signaling and plays a critical role in B cell activation and the formation of immunological memory. CD95 is well known for its role in promoting cell death and is induced in B cells after activation [25]. Accordingly, due to B cell death and the disappearance of B cell clones, studies have shown a decreased BCR repertoire in immune non-responding PWH [26]. Important B cell subsets, their phenotypic markers are listed in Table 1. The Table also shows effects of HIV infection and ART on these subsets. 

Primary HIV infection induces intense polyclonal activation of B cells in virus-infected individuals. The activation results, at least in part, in increased production of several pro-inflammatory mediators from innate immune cells. The mediators include type I interferons IL-6, TNF-α, IL-15, IL-1β, IL-18, B cell-activating factor (BAFF; also known as the B Lymphocyte Stimulator or BLyS), A proliferation-inducing ligand (APRIL) and others [46]. BAFF and its analog APRIL have a special role in B cell biology. BAFF is a member of the TNF superfamily-13b (TNFSF-13b), and both its membrane-bound and soluble forms are overproduced in HIV infection. The increased BAFF concentrations in the circulation correlate with disease progression in HIV and SIV infections. BAFF promotes polyclonal B cell activation and the appearance of the CD19^+^IgM^+^CD1c^+^CD27^+^ CD21^low^CD10^+^ B cell subset, termed as precursors of MZL B cells in HIV-infected individuals [47]. It also contributes to the survival of transitional/immature B cells, which numbers increase in HIV-infected individuals (Table 1). BAFF also promotes extrafollicular differentiation and the maturation of B cells, promoting the production of low-affinity antibodies and decreased numbers of MBC and LLPC [47]. In addition, it rescues autoimmune B cells from peripheral deletion, leading to the appearance of autoantibodies in HIV-infected individuals. On the other hand, the production of APRIL (TNFSF-13a) that promotes the survival of PC decreases in HIV-infected individuals, and its levels in the circulation correlate with slow disease progression [48]. Furthermore, increased gut permeability in HIV-infected individuals also leads to the translocation of microbial products such as LPS into the circulation. LPS activates immune cells, including B cells [49]. The polyclonal activation of B cells results in the loss of germinal centers (GCs), driving extrafollicular maturation and differentiation of B cells [14,19,50,51]. This shift from the follicular (FP) to extrafollicular pathway (EFP) contributes to generating tissue-like memory B cells (TL-MBCs) that are exhausted and less functional [35]. In the context of HIV infection, the role of the EFP response is of critical value, as they can result in a hyperactivated but less effective humoral response in PWH. This reduces the production of high-affinity antibodies and the generation of long-lived MBC and, hence, long-term immunity. These EFP responses contribute to the reduced vaccine responses observed in PWH [35].

The HIV-driven polyclonal activation of B cells occurs very early in the course of the infection. Very early in the HIV pandemic history, researchers reported increased numbers of spontaneously antibody-secreting cells (ASCs). The polyclonal activation results in the activation of multiple B cell clones irrespective of their specificity, leading to increased gammaglobulinemia, autoantibodies and B cell turnover, as well as decreased proliferative responses to T cell-independent (TI) antigens compared to HIV-negative healthy individuals [19,52,53]. The decreased responses to TI antigens in these patients increase their susceptibility to pneumococcal and other bacterial infections.

A significant reduction in the proportion of unique B cell receptor clonotypes within both naïve and memory B cell populations in the immune non-responder group was observed compared to immune responders, suggesting a diminished diversity of the B cell receptor (BCR) repertoire in HIV immune non-responders [26]. Altered expression of genes related to terminal differentiation and IFN stimulation were noted in B cells from viremic patients. CD21^low^CD95^+^ B cells increased in these patients. They also had a higher expression of B cell maturation antigen (BCMA) and lower expression of BAFF receptor (BAFFR) as compared to B cells from aviremic HIV-negative controls. It is noteworthy that both BCMA and BAFFR are activated by BlyS, the former receptor being a poorer responder than the latter one. Cumulatively, these alterations result in increased susceptibility of B cells from viremic patients to cell death. They also significantly impair the B cells’ ability to respond effectively to antigenic stimulation [54].

HIV induces significant quantitative and qualitative perturbations in different B cell subsets (Table 1). They are the following:

**Immature/Transitional (IT) B cells:** They are immature B cells that have just exited the bone marrow and transit to secondary lymphoid organs (SLOs) such as the spleen and lymph nodes through blood. In the SLO, IT differentiate into mature naïve B cells [10]. Normally, they comprise ~5% of B cells in the circulation. Due to higher cell turnover rate, their frequencies increase in the early course of HIV infection, the increase correlating with circulating levels of IL-7 [55,56,57]. The cells are very efficient in transmitting HIV to CD4^+^ T cells. The infected individuals with higher frequencies of IT B cells early in the course of the infection are more likely to experience rapid disease progression [58]. Their frequencies correlate negatively with CD4^+^ T cell counts [5,27].

**Naive B cells:** They circulate in the peripheral blood and lymphoid tissue and migrate to regional lymph nodes after encountering antigen. Their numbers are reduced in HIV-infected individuals due to enhanced apoptosis. Furthermore, they become activated and exhausted in HIV-infected individuals. Within the naïve B cell compartment, relative percentages of resting and activated naïve B cells are decreased and increased, respectively, in HIV-infected individuals compared to HIV-negative healthy controls (Table 1) [33]. A significant proportion of naïve B cells in HIV-1 infection exhibit reduced CD21 levels and a diminished response to B cell receptor stimulation. Single RNA sequencing showed reduced differentiation and abnormal cell cycles in naïve B cells from HIV-infected viremic and immune non-responding PWH, despite effective suppression of HIV replication [26].

**Marginal Zone-Like (MZL) B cells:** Humans have innate-like B cells, and due to their phenotypic and functional resemblance with murine marginal zone (MZ) B cells, they are called MZ-like (MZL) B cells. MZ B cells are well defined in rodents and primates and reside in the marginal zone at the interface between non-lymphoid (red pulp) and lymphoid (white pulp) regions of the spleen, gut-associated lymphoid tissue (GALT) and mesenteric lymph nodes. Murine MZ cells do not recirculate in blood, whereas MZL B cells do recirculate in blood between the spleen and GALT in humans [59]. They originate from B cell progenitors in the bone marrow as IT cells. MZL B cells express Notch-2 and interact with the IT ligand Delta-like-1 present on the surface of marginal zone stromal cells. Interestingly, MZL B cells may develop into memory-type cells without exposure to antigens. At a steady state, they produce natural IgM antibodies for housekeeping functions such as clearance of apoptotic and dying cells. These antibodies are weakly self- and/or polyreactive and are produced without stimulation by a foreign antigen [60]. MZL B cells are important for protection from blood-borne encapsulated bacteria such as Pneumococcus pneumoniae and respond to non-protein polysaccharide vaccines in a T cell-independent manner through the EFP (Figure 1). Upon contact with the bacteria/bacterial antigen, they rapidly differentiate into short-lived plasma cells that secrete low-affinity and less diversified antibodies [39]. To induce high-affinity antibodies and hence protection against bacterial infections, microbial capsular polysaccharides are conjugated with a carrier protein. These ‘conjugate vaccines’ activate B cells, engage TFH and elicit high-affinity antibody responses against bacterial infections [12,13].

In humans, CD27^+^CD21^+^CD1c^++^IgD^+^IgM^+^ B cells are considered MZL B cells [61]. Similar to naïve B cells, MZL B cells also show a decreased expression of CD21 in HIV-infected individuals. Their total numbers are also decreased in these individuals [40]. However, the relative frequencies of CCR6-expressing MZL B cells are significantly increased in HIV-infected individuals. Responding to the CCR-6 ligand (CCL-20), the cells migrate to peripheral mucosal niches [33,56]. The decreased and exhausted phenotype of MZL B cells makes HIV-infected individuals more susceptible to bacterial infections. MZL B cells are efficient antigen-presenting cells. The simultaneous stimulation of MZL B cells with a self-antigen with repetitive epitopes and a TLR ligand activates them for producing autoantibodies. Under these conditions, they express CXCR5; enter B cell follicles; present self-antigens to FDC and TFH; initiate GC reactions against self-antigens and contribute to autoimmune diseases such as systemic lupus erythematosus (SLE), Sjögren’s syndrome (SS) and rheumatoid arthritis (RA) [60].

In the literature, confusion exists between IgM^+^ memory B cells and MZL B cells. The former cell type shows SHM in their BCR represent and FBC that have exited GC reactions without undergoing a class switch. On the other hand, MZL B cells (IgM^++^IgD^+^) differentiate from IT through Notch signaling; express TLR2, 4 and 9 and become activated through TLR ligands and remain antigen-naive [62,63]. The IgM^+^ MBC can be distinguished from MZL B cells, as the former cells undergo somatic mutations in the Bcl6 gene, whereas the latter cells express high levels of CD1c; the Bcl6 mutations occur only in MBC that have undergone GC reactions [64]. The two cell lineages reside in distinct microanatomic locations in the spleen and GALT [65].

**Plasmablasts (PBs):** They are antibody-secreting short-lived dividing B cells that survive only for 3–5 days [67]. Phenotypically, they are CD19^+^CD20^−^CD38^++^. They are produced very early in the course of an infection or vaccination. They reach peak numbers in 10–14 days in the circulation after primary vaccination and in acute infection. However, they appear earlier upon recall responses and are short-lived. Their frequencies decrease in chronic infection [27,52,68]. They are responsible for the initial wave of antibody production in an infection or vaccination [67]. Antigen-activated B cells develop and differentiate into plasmablasts when they receive little or no help from TFH cells, do not enter GC, and therefore, they undergo limited SHM and class switching. They provide immediate protection from invading pathogens before the production of high-affinity antibodies through GC reactions. PB comprise a heterogeneous population: they may originate from activated naïve or activated memory B cells through EFP with or without T cell help [31]. Recently, it was shown that a majority of antibody-secreting PBs have exited cell cycles, despite expressing Ki67, and are really not ‘blasting’, so calling them PBs is a misnomer [69]. It would be more appropriate to call them early effector MBCs. Interestingly, PB can also arise in response to antigen non-specific innate stimuli such as cytokines, thus increasing their relative percentages under inflammatory conditions [68].

Plasmablasts secrete IgG or IgA antibodies, while some may be more like memory B cells. A small fraction of them migrates in response to CXCR4 and CXCR3 ligands to the bone marrow and spleen and differentiates into PC [69,70]. They have been exploited to isolate therapeutic antibodies. Interestingly, frequencies of plasmablasts in the circulation of individuals vaccinated with two doses of SARS-CoV-2 mRNA vaccine correlate with antibody titers six months post-vaccination, suggesting their importance in antibody responses [68].

**Memory B cells (MBCs):** Typical MBCs are CD27^+^. In general, HIV induces exhaustion and activation in the MBC compartment. They are classified into switched and non-class switched MBC. The classification is based upon whether they have undergone class switching, i.e., they have switched their sIg expression from IgM to IgG or IgA. It is noteworthy that IgE^+^ MBC have never been described in humans. IgE-secreting PC cells probably arise from IgG+ MBC when they re-enter GC, undergo class switching and differentiate into IgE-secreting PC. Switched MBC (SMBC) arise from GC reactions in response to T cell-dependent antigens. On the other hand, non-switched MBC (NS-MBC) continue expressing IgM with or without low levels of IgD. IgM^+^ MBC are reduced in HIV-infected individuals and their numbers are not normalized by ART in chronic HIV [41]. A rare population of IgD^+^IgM^−^ (IgD only) MBC have also been described in humans; little is known about these NSMBC in HIV-infected individuals [71].

Atypical memory B cells do not express CD27, the classical marker for typical MBC. Phenotypically, they are CD27^−^IgD^−^CD21^low/−^ and are also called double-negative (DN), age-associated, late or exhausted MBC. They develop from switched MBC after chronic antigen stimulation, from follicular B cells that exit prematurely from GC or from the follicular B cells that differentiate through EFP [72]. They express a variety of inhibitory molecules such as FcRL4 (a receptor for Fc region of IgA), FcRL5 (a receptor for Fc region of IgG), CD32b (FcγRIIb; an inhibitory receptor for Fc region of IgG), Sialic acid binding immunoglobulin-like lectin-6 (SIGLEC6), CD95, etc. Based upon the level of expression of CD21, CXCR3, CXCR5, T-Bet and CD11c, atypical MBC have been divided into different subsets that serve distinct yet overlapping roles [73].

A subset of atypical MBC that are phenotypically CD27^low^CD20^++^CD21^low^FcRL4^+^ was first identified in human tonsillar tissues and named tissue-like MBC (TL-MBC) [74]. TL-MBC are expanded in HIV-infected viremic individuals. Several investigators have noted increased frequencies of these cells in the circulation of individuals with HIV viremia [40,43,75]. FcRL4 expression is notably higher in B cells from HIV-viremic individuals compared to those from HIV-aviremic and HIV-negative individuals. The expression is particularly prevalent in B cells with a tissue-like memory phenotype compared to B cells with classical memory (CD27^+^) or naive (CD27^−^CD21^++^) phenotypes. Interestingly, this exhausted B cell subset was enriched for HIV-specific but not for influenza-specific B cells [43,75]. Their frequencies also increase in other chronic infections, such as lymphocytic choriomeningitis virus (LCMV), Hepatitis C (HCV) and malaria, as well as in autoimmune diseases that are accompanied by increased chronic immune activation and inflammation [76]. TL-MBC have decreased frequencies of SHM, and the antibodies secreted by them have a decreased capacity to neutralize the virus. They have a higher mutation level in their IgV_H_ genes than in naïve and NSMBC but lower than in SMBC. The cells show poor proliferation in response to B cell stimuli, most probably due to their high expression of many inhibitory receptors [19,23,43,63,75,76]. In viremic patients, HIV-specific B cell responses are enriched in activated and exhausted memory subsets but in resting ones in aviremic individuals [27,38]. Atypical MBC or age-associated B cells are found in increased frequencies in aged mice and humans and contribute to inflammation, cancer and autoimmunity [77].

**Plasma cells (PCs)**: Phenotypically, bone marrow PC are CD19^+/−^CD20^−^CD38^+^CD138^+^, whereas tonsillar PC are CD19-CD20+CD138^+/−^ [78]. They are terminally differentiated quiescent effector B cells that constitutively produce antibodies. PC are rarely found in peripheral blood. They express CXCR4 and CXCR3 and migrate to and reside in the bone marrow, spleen and lamina propria of mucosae. In the bone marrow, PC become short-lived (SLPC) or long-lived PC (LLPC); SLPC live for months, whereas LLPC live for decades or for life. The LLPC provide long-term protection by constitutively secreting antibodies [70]. The factors determining PC longevity remain unknown. LLPC express higher levels of CD138 and BLIMP-1 [79]. The persistence of high-affinity vaccine-specific antibody titers is an indirect measure of the longevity of the vaccine-induced MBC. Little is known about PC in PWH, as they are located in tissues and require survival cues from their niches. However, there is a high incidence of plasma cell disorders in PWH that can range from polyclonal hypergammaglobulinemia to monoclonal gammopathies and multiple myeloma; the causes of these disorders include immune activation, immunodeficiency and inflammation [80]. Additionally, IgA^+^ plasma cells, which are the predominant output of intestinal germinal centers, were found to be reduced during viremic HIV infection (Table 1) [55,81].

**Regulatory B cells (Bregs)**: Bregs modulate immune responses through the production of anti-inflammatory cytokines such as IL-10, IL-35, IL-37 and TGF-β. At least in some human disease conditions, Bregs were found to express FoxP3, FasL and PDL-1 [82,83]. Importantly, Bregs are implicated in supporting the differentiation of regulatory T cells (Tregs) and in maintaining immune tolerance. They can also modulate vaccine efficacy by influencing the balance between immune activation and tolerance [83,84,85]. In healthy individuals, Bregs constitute <1% of the total B cells. Their percentages increase in HIV-infected individuals (Table 1), where they contribute to immune dysfunction associated with HIV infection by impairing T cell function, primarily through IL-10 production and potentially through PD-L1 expression. Compared to Bregs from healthy individuals, a significantly higher percentage of Bregs in PWH spontaneously express IL-10. Furthermore, Breg-mediated suppression of CD8^+^ T cell proliferation was also found to be IL-10-dependent, as the in vitro depletion of Bregs resulted in enhanced proliferation of both total CD8^+^ T cells and CD8^+^CD107a^+^ T cells (that have undergone release of their cytotoxic granules) [86]. Studies have reported that Breg frequencies in HIV-infected individuals correlate positively with the viral load and negatively with CD4^+^ T cell counts, the two markers of disease progression [44].

Interestingly, another subset of Bregs that express granzyme B (GZB) has also been described in HIV-infected individuals, autoimmune diseases, cancer patients and organ transplantees. The cells produce Lymphotoxin-A (LTA) in addition to IL-10 upon activation. These Bregs also inhibit CD4^+^CD25^−^ effector T cell proliferation through LTA [87]. The concurrent stimulation of B cells with IL-21 and CD40L is known to promote the development of PC, whereas, in the absence of CD40 stimulation, IL-21 promotes Bregs. In the case of HIV-infected individuals, it was demonstrated that Nef induces CD4^+^ T cells to produce IL-21 without the induction of CD40L [88]. The Nef-stimulated CD4^+^ T cells activate B cells through IL-21 without CD40 stimulation and convert the latter cells into GZB-expressing Bregs [88].

## 4. Impact of Anti-Retroviral Therapy (ART) on B Cells in PWH

ART is a combination of different anti-retroviral drugs used to manage HIV, and although it cannot cure HIV, it effectively suppresses viral replication, potentially lowering the viral load in the body to undetectable levels. Suppressed viral levels prevent the destruction of CD4^+^ T cells and restore their counts and functionality towards normalcy. They also reduce sexual transmission of the virus [89]. ART reverses HIV-induced perturbations in frequencies of different B cell subsets. It results in the reduction in numbers of plasmablasts, immature/transitional B cells, TL-MBC and activated naïve and activated memory B cells while increasing the numbers of resting naïve and resting memory B cells [27] (Table 1). The extent of the ART-induced normalization in the numbers and frequencies of different B cell subsets depends upon the time when it is started following HIV infection. If started late during chronic infection, ART cannot fully normalize the virus-induced changes, as the damage to the B cell compartment has already been irreversibly done. Even after one year of ART in chronically infected individuals, higher numbers of immature/transitional B cells persist. On the other hand, starting ART early in the infection normalizes B cell perturbations; however, despite normal numbers, B cells show a heightened state of activation and exhaustion [27,90]. ART also partially reverses HIV-altered B cell function. B cell proliferation and differentiation in response to HIV and non-HIV antigens improves. A partial reversal of HIV-induced B cell dysfunction can be observed within a few weeks of initiating antiviral therapy.

At the onset of primary HIV-1 infection, decreased numbers of B cells, IgM-positive memory B cells and hyper-gammaglobulinemia were noted. B cells showed a reduced expression of CD25 upon their activation by autologous T cells. These defects normalized after six months of antiretroviral therapy [28]. In HIV-infected children, the impact of ART on the B cell compartment can be attributed to the suppression of HIV replication and decreased viremia [19]. ART significantly decreases hyper-activation, exhaustion, turnover and apoptosis in B cells. It also significantly decreases the frequency of IgG-secreting B cells in both acutely and chronically infected individuals. In contrast, the hyper-gammaglobulinemia typically seen during chronic infection resolves more slowly. The reductions in IgG-secreting B cells and hyper-gammaglobulinemia continue as long as HIV-1 replication is controlled. ART also reduces IgG-secreting B cells specific for HIV antigens such as gp120 and gag proteins, resulting in decreased HIV-specific antibodies. The reduction happens simply due to the non-availability of these antigens caused by viral suppression [91].

Important prognostic indicators of B cell perturbations included viremia and nadir CD4^+^ T cell counts. Despite effective ART, resting memory B cell depletion in chronic HIV infection is not fully reversed (Table 1) [92]. ART increases total circulating B cells back to normal numbers, primarily due to rise of naïve B cell through improving B cell output from the bone marrow. However, 8 weeks post-ART, a complete recovery of the switched memory B cell subset was not achieved, even though there was a reduction in the CD21low B cell population [93]. In children <5 years of age, after 12 months of ART, TL-MBC, MBC and activated memory B cells remained significantly higher than in healthy controls, indicating these subpopulations did not fully normalize. Additionally, the frequencies of switched/non-switched memory (SMBC/NSMBC), and resting memory B cells were still lower compared to healthy controls, suggesting incomplete recovery of the B cell compartment even with treatment [94]. Another study in children that were vertically infected with HIV showed that, despite the apparent normalization of clinical and phenotypic parameters due to effective ART, qualitative defects still persist in B cells. After >2 years of ART, the children’s CD27^+^CD21^−^ activated memory B cells had increased the expression of 28 genes compared to those of healthy controls [7]. The genes were for encoding proteins belonging to the inflammatory cascade and B cell activation. Not surprisingly, the children responded poorly to influenza vaccination. Effective ART helps restore the normal distribution of peripheral naive and MZ-like B cells; however, IgM^+^ MBC (classical non-switched MBC, often confused with MZL B cells) are not normalized [33].

In summary, the effects of ART on B cells are primarily due to the reduction in HIV viraemia and increase in CD4^+^ T cells counts, and they are linked to a decrease in the frequency of immature/transitional B cells. Normalization of B cell counts and of B cell subpopulations, especially the increasing of naïve and resting memory B cells, may explain the improved humoral immunity after ART treatment [29]. ART-induced increases in B cell numbers are linked to a reduction in the frequency and quantity of B cells susceptible to cell death, particularly activated mature CD21low B cells and immature transitional B cells [19,89]. While most B cell defects in HIV infection can be reversed by antiretroviral therapy, a notable exception is the persistent loss of memory B cells and the reduction in memory B cell function observed in individuals with chronic HIV infection [19]. Following antiretroviral treatment, an increase in the CD4^+^ T cell count is observed alongside a stepwise decrease in Breg frequency [44].

## 5. CD4^+^ T Cell Help for B Cells in PWH

As stated above, TFH are essential for GC reactions in which antigen-activated B cells undergo somatic hypermutations in their BCR, affinity maturation and class switching. Phenotypically, TFH are CD4^+^CXCR5^+^ICOS^+^PD-1^+^Bcl6^+^; CXCR5 promotes their migration to B cell follicles in response to CXCL-13 [15]. Interactions between cognate TFH and B cells provides co-stimulatory signals to B through CD40-CD40L interaction and cytokines (IL-21, IL-4, CXCL13 and IL-17) [15]. In particular, IL-21 from TFH induces the expression of AID required for SHM and affinity maturation, as well as class switching.

Boswell et al. [95] reported decreased frequencies of cTFH in the circulation of ART-naïve PWH. However, it was very surprisingly noted that both HIV-specific and HIV-non-specific TFH in the lymph nodes of PWH were expanded [57]. The frequencies of Gag-specific THF were the highest. It was found that viremia drives the expansion of these cells. The expansion was associated with increased numbers of GC B cells and plasma cells and with increased IgG1 production [57]. Another study [36] noted that expansions of cTFH in ART-naïve HIV-infected individuals correlated positively with the plasma HIV-1 viral load and negatively with CD4^+^T cell counts in acute HIV infection. Their results showed that HIV-1 infection drives the expansion of cTFH cells, which, in turn, leads to perturbations of B cell differentiation through the inducible co-stimulatory molecule (ICOS) signaling during the acute stage of the infection [36,96]. It is noteworthy that, despite suppressive ART, lymph node DC harbor replication-competent HIV. These latently infected DC may be compromised in inducing the differentiation of CD4^+^ T cells into TFH [97]. Furthermore, follicular dendritic cells (FDCs), the stromal cells found in primary B cell follicles and germinal centers of secondary follicles, retain virions on their surface and may cause infection and dysfunction of TFH. The process may be hastened by the exclusion of CD8^+^ T cells from the follicles [98,99].

It has been shown that GC TFH and B cells express higher levels of PD-1 and PDL-1, respectively, in PWH. Their interaction inhibits TFH help for B cells. Thus, despite their expanded numbers, qualitative changes in the TFH in PWH results in decreased frequencies and the poor quality of CD27^+^IgD^low^ MBC [100]. Furthermore, the cells were shown to be compromised in their ability to produce IL-4, an important cytokine required for B cell function, development and differentiation [101]. Comparing transcriptional profiles of GC TFH after their in vitro interaction with GC B cells from PWH and HIV-negative individuals, Chakhtoura et al. [102] showed that TFH from people with chronic HIV infection were impaired in their ability to transcribe c-Maf due to attenuated expression of IL-6R and STAT-3. They also showed that GC TFH from PWH had reduced expression of adenosine deaminase (ADA)-1; the supplementation of ADA-1 considerably rescued c-Maf in the GC TFH from HIV-infected individuals. The authors further showed that GC TFH from HIV-infected individuals were compromised in their ability to interact with, and provide help to, GC B cells. These defects in the GC TFH of HIV-infected individuals may be responsible for reduced numbers of GC, impaired anti-HIV antibody responses and early death of HIV-specific MBC.

It was demonstrated that PD-1^+^ TFH are highly susceptible to HIV infection. Tissue-resident TFH constitute the main cellular sanctuary for latent HIV in the body. At least in part, this reservoir may persist due to the reason that CD8^+^ T cells rarely infiltrate GC, and even they do so, they are less cytotoxic than their counterpart cells present in the circulation [99,103]. In PWH receiving ART, CXCR3^−^PD-1^+^ TFH are also the main cell type in the peripheral blood among CD4^+^ T central memory cells that harbor replication-competent HIV [104]. TFH can be classified into CXCR3^+^CCR6^−^ TFH-1 that produce IL-21 and IFN-γ, CXCR3^−^CCR6^−^ TFH-2 that produce IL-21 and IL-4 and CXCR3^−^CCR6^+^ TFH-17 that produce IL-21 and IL-17A. The three subsets preferentially promote class switching towards IgG, IgE and IgA, respectively. A small subset of TFH express CXCR3 and CCR6, secrete both IFN-γ and IL-17, and are called TFH-1/17 [13].

A subset of CD4^+^ T cells, called Follicular Regulatory T (TFR) cells, has the same phenotype as TFH, but they express FoxP3 and CTLA-4 (two phenotypic markers of the immunosuppressive Tregs) but not Bcl6. They inhibit and neutralize TFH functions in HIV-infected individuals [105]. Many studies investigating TFH in HIV-infected individuals did not distinguish between TFH and TFR. It was recently demonstrated that the frequencies of TFR, as opposed to those of TFH, do not increase in HIV-infected individuals [106]. The authors showed that, compared to TFH, TFR express higher levels of CCR5, a chemokine receptor that serves as a major HIV co-receptor. The study raises the possibility that TFR, through constitutive expression of CCR5, may harbor latent HIV and contribute towards the viral reservoir. Together with TFH, they constitute the CD4^+^ follicular T cells [107].

Unlike TFH, TFR do not produce IL-21. Rather, they produce cytokines such as TGF-β and IL-10 and dampen early GC reactions and the production of autoreactive antibodies [108]. As mentioned above, the early disruption of GC reactions leads to the emergence of CD27^−^IgD^−^CD21^−^ atypical MBC [72]. An optimum TFH/TFR ratio in the blood of an individual corresponds to their ability to generate robust T cell-dependent antibody responses to vaccines without eliciting autoreactive antibodies. Studies have shown unusually increased numbers of TFH and/or a decreased TFR/TFH ratio in autoimmune diseases such as systemic lupus erythematosus (SLE) and Sjogren’s syndrome [107]. A unique regulatory subset of CXCR5^++^CD44^++^CD8^+^ T cells was demonstrated in chronic SIV-infected macaques. The cells migrate to follicles and inhibit IL-21 production from TFH through TIM-3 and, consequently, virus-specific MBC and PC [105). Their presence in PWH has not been reported.

Early ART and persistent viral suppression normalize the TFH number; however, they still harbor functional defects and are more activated [108]. The functional defects result in suboptimal TFH help for B cells, resulting in extrafollicular maturation of B cells. It has been demonstrated in PWH vaccinated with a quadrivalent split virion inactivated influenza vaccine that antibody responses in the plasma and saliva correlated with frequencies of cTFH [109]. There is a need to learn more as to the effects of HIV and ART on TFR numbers, phenotype and functions.

## 6. PWH’s Responses to COVID-19 Vaccines

Persistent HIV-induced perturbations and functional defects in B and TFH compartments, despite suppressive ART, contribute to decreased antibody responses to commonly used vaccines in PWH compared to HIV-negative healthy individuals [42,110]. In the following section, we will discuss the immune responses of PWH to anti-COVID-19 vaccines, focusing on the dynamics of B cell subsets and TFH concurrent with the humoral responses.

Severe Acute Respiratory Syndrome Coronavirus-2 (SARS-CoV-2), the causative agent of the Corona Virus Induced Disease-2019 (COVID-19), emerged in late 2019 and spread all over the world within months. As per the WHO data, up to September 2024, more than 775 million people have been infected with the virus, and more than 7 million of them have died (https://data.who.int/dashboards/covid19/cases, accessed on 18 September 2024). The pandemic created a sense of great urgency for developing a safe and effective anti-COVOD-19 vaccine. Among several types of vaccines, the viral protein Spike (S) mRNA-based vaccines (mRNA-1273 and BNT162b2), viral vector-based vaccines such as ChAdOx1-nCoV-19 and killed whole virus-based vaccines such as CoronaVac have been developed and extensively used to vaccinate PWH and the general population all over the world [111]. When the vaccines were first introduced, PWH were designated as a priority population to receive them because of their higher vulnerability to the infection and hospitalizations. It has been concluded that the vaccinations, although they do not prevent breakthrough infections, greatly reduce the severity of the disease and hospitalization of the vaccinated individuals. The virus, despite vaccinations, is spreading due to the continuous emergence of thousands of immune-escaping variants named by the WHO by Greek alphabet letters Alpha, Beta, Gamma, Delta, Omicron and so on, each with several sub-lineages, such as BA.4, BA.5 and XEC for Omicron [112,113]. With the emergence of novel, more contagious variants, known as variants of concern (VOC), it has become a challenge to induce protective immunity through vaccinations.

Initial studies showed comparable antibody responses in PWH and HIV-negative controls following a natural infection with SARS-CoV-2 infection [114,115], although, in one study, it was shown that SARS-Cov-2-specific T cell responses were delayed in PWH, who had detectable viral load and were immunosuppressed despite ART [115]. However, Krause et al. [35] showed that, in SARS-CoV-2-infected PWH, B cell responses are skewed towards the extrafollicular pathway. The authors investigated B cell subsets, their homing, maturation and regulatory features longitudinally between the two cohorts, and found increased expression of PDL-1 on MBC in PWH with a reduced homing capacity to GC and showed delayed and reduced GC reactions. PWH had increased frequencies of activated DN memory B cells and activated naïve B cells. The extrafollicular responses in PWH correlated positively with their viral loads and negatively with the CD4^+^ T cell counts [35]. It is noteworthy that extrafollicular responses are rapid but give rise to lower-affinity antibodies and memory responses that do not last long and cannot evolve to neutralize new variants. The authors found decreased expression of GC homing markers, CD62L and CXCR5, on SMBC in PWH. Activated naïve (CD95^+^) and DN MBC were found to differentiate through the GC-independent EF pathway. In PWH, DN cells did not express CD21 and CXCR5 but did express CD11c and CD95, typical markers of TL-MBC [35,116].

Following one or two doses of different vaccines (mRNA vaccines, adenovirus vectored vaccine or killed whole virus vaccine), several researchers compared the titers of receptor-binding domain (RDB)-specific antibodies, Spike (S)-specific and virus-neutralizing antibodies (Table 2). It is noteworthy that SARS-CoV-2 surface protein S binds with its receptor, the angiotensin converting enzyme-2 (ACE2), and initiates the infection process in susceptible host cells through the receptor binding domain (RBD) present in the viral S protein, and RBD-binding antibodies that inhibit this attachment neutralize the virus [117]. The results from several studies (listed in Table 2) showed that antibody responses were lower and declined earlier in PWH as compared to HIV-negative controls [118,119]. The antibodies from the vaccinated PWH were less efficient in neutralizing SARS-CoV-2 variants such as RBD with D614G (a mutation that was first noted in the RBD in SARS-CoV-2 that increases its transmissibility and is found in subsequent variants) and Delta (B.1.617.2). The responses were particularly compromised in PWH with low CD4^+^ T cells counts and unsuppressed viral loads [118,119,120,121,122,123,124]. Polvere et al. [125] investigated antibody functionality and MBC phenotypes in PWH vaccinated with two doses of mRNA vaccines. The PWH had different CD4^+^ T cell counts, ranging from <350 to >500. The authors showed that PWH with lower CD4 counts had lower titers than those with higher counts; however, both PWH and HIV-negative controls had similar RBD/ACE2-blocking antibodies, and these antibodies decreased more rapidly in PWH. S-specific IgG and IgM titers were initially similar (at two months post-second dose); however, they declined more rapidly in PWH and, at 5 months post-vaccination, were lower in PWH. Importantly, PWH showed lower S-specific SMBC (CD27^+^IgD^−^) and had higher DN MBC with an exhausted phenotype [125].

These investigations led to recommendations of using three or more vaccine doses of the vaccines in PWH and other immunocompromised individuals. Following these recommendations, several studies have investigated antibody responses in PWH following three doses of anti-COVID-19 vaccinations. In these studies, a third dose with mRNA vaccines was shown to increase S-specific antibody titers, as well as their increased maintainability in PWH, narrowing the gap between PWH and HIV-negative controls that existed following the second dose [127]. The third dose with an inactivated whole virus vaccine, Sinovac, was shown to increase neutralizing antibody titers, expand CXCR3^+^ TFH and increase the maturity and potency of the vaccine-specific antibodies [130]. The authors also showed that CXCR3^+^ TFH were superior to their CXCR3^−^ counterparts for maintaining GC reactions and inducing the differentiation of B cells into ASCs. They suggested that a stronger induction of CXCR3^+^TFH by additional vaccine doses could provide better and longer-lasting protection against the virus [130]. A third dose of vaccination with mRNA-based vaccines was shown not only to narrow the gap between PWH (irrespective of their CD4^+^ T cell counts) and HIV-negative healthy controls but also increased antibodies specific to Omicron variants in PWH, although these titers remained lower as compared to healthy controls. The authors also showed that the mRNA-1273 vaccine induced better responses than BNT162b2, which were not influenced by low CD4^+^ T cell count nadir [128].

Only a few studies have investigated the dynamics of B cells or of TFH in antibody responses to mRNA vaccines in PWH and compared them with those in HIV-negative controls. In a seminal and comprehensive study, Touizer et al. [37] showed that PWH developed delayed and lower neutralization titers following three doses of anti-COVID-19 vaccines compared to HIV individuals. The authors ascribed these lower and delayed responses to global defects in their MBC. Furthermore, PWH were shown to have lower levels of S-specific cTFH in their circulation. The vaccinated PWH with a delayed/low neutralization response had increased frequencies of atypical (CD27^−^CD21^−^) and activated (CD27^+^CD21^−^) MBC but lower frequencies of resting (CD27^+^CD21^+^) MBC. They showed that lower frequencies of neutralizing antibody titers correlated with lower frequencies of resting MBC. The third dose of the vaccine increased the breadth of neutralization against the Omicron variant (BA.1), but the neutralization titers still remained lower as compared to HIV-negative individuals. The MBC from the vaccinated PWH underwent reduced class switching after the third dose and had more S-specific IgM^+^ MBC and less IgG^+^ MBC than the second dose. Importantly, they had decreased frequencies of CXCR3^+^CXCR5^+^ TFH [37]. Surprisingly, PWH with low or absent neutralizing antibodies had detectable variant cross-reactive T cell responses and showed an expansion of CXCR3^+^CD127^+^CD8^+^CD38^+^CCR7^+^CD45RA^+^ T cells in their circulation. The authors reported that following the first dose, over 82% of controls developed neutralizing antibodies (NAs) compared to 29% in PWH; individuals with prior SARS-CoV-2 infection showed more robust NA titers irrespective of their HIV status; however, the titers were lower than in HIV- at all time points. PWH with no prior infection mostly had no detectable NA after the first and second dose, and the NA disappeared in most of them before administration of the third dose, while the third dose narrowed the gap between PWH and healthy controls. Interestingly, the authors [37] found no correlation between CD4 counts and NA titers; instead, they found a positive correlation between S-specific MBC and NA titers. The study implicates impaired MBC homeostasis and THF help in sub-optimal humoral responses to anti-COVID-19 mRNA vaccines in PWH.

With the anticipation that a fourth dose of vaccination may induce durable variant-specific humoral responses in PWH, a few researchers have investigated this possibility [126,129,131]. They concluded that the fourth dose of anti-COVOD-19 vaccines in COVID-naïve PWH results in a transient reactivation of pre-existing S-specific immunological memory. It also enhances neutralizing antibody (NA) titers against WT, as well as against Omicron variants more than the third dose. However, in COVID-19-experienced PWH, the fourth dose only marginally increases NA titers against the WT virus but substantially increases the antibody responses against variants compared to COVID-19-naïve PWH underlining increased ‘hybrid immunity’; the immunity induced by the vaccine, as well as from the previous SARS-CoV-2 infection. Despite the fourth dose, the variant-specific NA titers remained lower than WT-specific ones irrespective of COVID-19 experience [126,129].

## 7. Potential Strategies for Improving Vaccine-Induced Immune Responses

Based upon past experience of vaccination for COVID-19 and other infectious diseases in PWH, the following suggestions can be made for improving vaccination outcomes in PWH:

**Starting ART early:** It has been well documented that an early start of ART and persistent suppression of viral replication is the key to maximally normalizing the defects in the B cell compartment [27,90]. This may be a challenge in resource-poor countries where access to ART is limited. In this context, the development of long-acting antivirals such as injectable Cabotegravir-Rilpivirine (administered every 1–2 months) will be very helpful in managing HIV [132].

**Optimizing vaccination strategies:** For SARS-CoV-2 vaccines, a longer interval between the first and second dose induces more robust antibody responses [133]. Furthermore, it was also shown that combining heterologous vaccines for priming and boosting increases antibody titers in response to vaccination for SARS-CoV-2 infection [134]. Increasing the number of booster doses to three or more induces better antibody titers in PWH. The antibody response to the high-dose influenza vaccine, approved for individuals aged ≥65 years, is superior to that of the standard-dose vaccine in PWH. A study done in the US found 50% reduction in influenza-like illness among PWH receiving a high dose quadrivalent influenza vaccine (recommended for adults aged 65 and above) compared to those receiving a standard low dose recommended for adults less than 65 years [135]. Similar results were reported in higher dose vaccination for H1N1; following vaccination, the high-dose group’s seroprotection rates for H1N1, H3N2 and influenza B were significantly higher [136]. The schedule narrowed the gap between HIV-positive and HIV-negative women in the same setting [137]. It was also demonstrated that the dosing schedules could affect the immunogenicity of vaccines. For example, in pregnant HIV-positive women, a double-dose regimen of an inactivated trivalent influenza vaccine demonstrated higher immunogenicity than a single-dose or two one month apart single-dose regimens [137]. Again, not all the studies are in accord; the high-dose influenza vaccine demonstrated greater immunogenicity compared with the standard vaccine in children with cancer but not in those with HIV [138].

**Countering inhibitory receptors on exhausted B cells:** The exhausted B cells have a unique inflammatory phenotype and produce pro-inflammatory cytokines such as IL-6. The inhibitory receptors also compromise ‘tonic’ ligand-independent BCR-induced pro-survival signals to B cells [139]. The upregulation of the inhibitory receptors impairs B cell functionality in PWH, resulting in reduced antibody production, altered memory responses and persistent low-grade inflammation, all of which adversely affect their responses to vaccines. It has been demonstrated that the functionality of exhausted B cells in PWH can be restored by inhibiting certain inhibitory receptors, such as FcRL4, FcRL5 or SIGLEC-6, through siRNA [76]. FcRL4 and FcRL5 bind FcR of IgA and IgG, respectively. Both activate SHP-1 and inhibit BCR-induced signaling. Their blockade through monoclonal antibodies concurrent with vaccination could be a useful strategy to enhance antibody responses in PWH.

**Restoring TFH effector functions:** As mentioned above, TFH become dysfunctional in PWH. It has been demonstrated that GC TFH in PWH lack the expression of adenosine deaminase (ADA), leading to dysregulated adenosine production. Exogenous ADA improves the B cell help of GC TFH and cTFH [140]. Interestingly, human TFH are very prone to ferroptosis, a form of programmed cell death mediated by the iron-dependent accumulation of lipid peroxidation products. It is countered by glutathione peroxidase 4 (GPX4), an important antioxidant enzyme and a selenoprotein. In this regard, selenium supplementation was shown to enhance GPX-4 and TFH function, as well as vaccine-induced antibody responses, in animal models [141]. Furthermore, the blockade of TNF-α was shown to restore IL-21 production from TFH and increase antibody responses to influenza vaccine in HIV-infected women [142]. Although the cytokine is needed to promote the formation of primary B cell follicles, high concentrations of the cytokine have been shown to deplete GC and TFH effector functions [143]. Further research is required to leverage the cytokine manipulation in enhancing GC reactions and TFH functionality in PWH.

**Neutralization of B cell-stimulating pro-inflammatory cytokines:** Although there are several pro-inflammatory mediators that induce polyclonal activation of B cells, BAFF excess in HIV infection is of a particular interest. It impairs humoral immune responses, causes loss of MBC and is associated with disease progression [47]. Its blockade with BAFF antagonists such as BR3-Fc may help in the normalization of dysregulated B cell development in PWH. Indeed, the blockade was shown to reduce GC inflammation and increase the frequency of SIV-specific MBC in Rhesus macaques [144].

## 8. Conclusions

- HIV causes intense polyclonal activation of B cells, resulting in hypergammaglobulinemia, perturbations and functional defects in all B cell subsets.
- It also causes the expansion of TFH; however, the expanded TFH become functionally defective in providing help to B cells.
- It causes global defects in memory B cells and induces the appearance of atypical memory B cells.
- The virus drives the maturation and differentiation of activated B cells via the extrafollicular pathway.
- ART normalizes these perturbations and restores cell functionality in TFH inadequately, especially when started late.
- Early ART restores most of the defects and perturbations in various B cell subsets; however, the cells remain in a heightened state of activation and exhaustion in PWH.
- Anti-COVID-19 vaccines are, in general, safe and well tolerated, as well as immunogenic, in PWH unless they are viremic and immune non-responders (CD4^+^ T cell counts < 350 per mm^3^).
- Three vaccine doses provide virus-neutralizing antibodies of higher magnitude, more durability and breadth (cross-reactivity with novel variants) as compared to two doses.
- Vaccination induces more robust humoral responses in PWH with prior exposure to SARS-CoV-2, highlighting the beneficial effects of hybrid immunity.
- Live attenuated vaccines are considered risky and are not recommended in PWH. However, in immune responders with improved immune status (CD4^+^ T cell counts >500 cells per mm^3^), potential benefits from such vaccines may outweigh the risks.
- Anti-COVID-19 vaccinations in PWH cause a transient increase in cell-associated viral RNA; viral reservoirs were only slightly increased in viremic participants [145,146,147].
- More fundamental research is needed to find novel ways of reducing inflammation, exhaustion and aberrant activation in B cell and TFH compartments in PWH.

## Figures and Tables

**Table 1:** **Effects of HIV infection and ART on B cell subsets**.

B Cell or Subset	Phenotype	HIV-Infected, No ART	Early ART	Late ART	Ref.
Total B cells	CD19^+^	↓	Normalized	Sub-normal	[27,28,29]
Activated/exhausted B cells	CD19^+^ CD80^+^ PDL1^+^ CD21^low^ FcRL4^+^	↑	Normalized	Above normal	[30]
Immature/Transitional	CD19^+^ CD27^−^ CD38^++^ CD24^++^ CD10^+^ IgM^+^ IgD^low^	↑	Normalized	Above-normal	[31,32]
Naïve (Resting)	CD19^+^ CD10^−^ CD27^−^ CD21^++^ CD24^+^ CD38^+^ IgM^low^ IgD^+^	↓	Normalized	Sub-normal	[32,33,34]
Activated Naive	CD19^+^ CD10^−^ CD27^−^ CD21^low^ IgM^+^ CD95^+^	↑	Normalized	Above-normal	[32,35]
Resting Memory	CD19^+^ CD10^−^ CD27^+^ CD21^++^ IgM^−^ IgD^−^	↓	Normalized	Sub-normal	[36,37]
Activated Memory	CD19^+^ CD10^−^ CD27^+^ CD21^low^ CD38^−^	↑	Normalized	Above-normal	[37]
Atypical Memory	CD19^++^ CD20^+^ CD21^low/−^ CD38^−^ CD27^−^ IgD^−^	↑	Normalized	Above-normal	[30,38]
Marginal Zone-like	CD19^+^ CD27^+^ IgM^+^ CD21^++^ CD23^−^ CD1c^++^	↓	Normalized	Subnormal	[39,40]
IgM-only Memory	CD19^+^ CD27^+^ IgM^+^ IgD^−^	↓	Normalized	Sub-normal	[27,41,42]
TL-MBC	CD19^+^ CD10^−^ CD27^−^ CD21^low^ CD20^+^ FcRL4^+^ CD11c^+^	↑	Normalized	Above-normal	[37,43]
Plasmablasts	CD19^+^ CD27^++^ CD20^−^ CD21^low^ CD38^++^ CD24^−^	↑	Normalized	Above-normal	[31,40]
Regulatory B cells	CD19^++^ CD25^++^ CD1d^++^ IL-10^+^ CD45RB^++^ PDL1^+^ CD24^++^	↑	Normalized	Above-normal	[44,45]
B cell activation	CD19^+^ CD25^+^	↑	↑	↑	[19]
Apoptosis prone	CD19^+^ CD95^+^	↑	↑	↑	[19]

The arrows ↑ and ↓ indicate frequencies above and below normal values, respectively. Signs: ++ indicates high expression, and low indicates low/weak expression. Immunoglobulins (Ig) shown are expressed on the cell surface.

**Table 2:** **Studies investigating the humoral immune response to COVID-19 vaccination in PWH**.

Year	Authors	Vaccine Type	Cohorts	Results & Conclusions	Ref.
2022	Nault et al.	mRNA; 1 dose	121 PWH, 21 HIV-healthcare workers (naïve to COVID-19	Anti-RBD IgG responses 3–4 weeks post-vaccination were lower in PWH with CD4 counts < 250; higher age associated with decreased immunogenicity	[118]
2022	Khan et al.	Ad26.CoV2.S; 1 dose	PWH with well controlled HIV, and HIV-controls with and without previous SARS-CoV-2 infection	Live virus neutralization response two months post-vaccination to Delta variant was not inferior in PWH with controlled viremia; viremic PWH had lower responses	[120]
2022	Cai et al.	Inactiva-ted SARS-CoV-2 vaccine; 2 doses	143 PWH and 50 HIV-healthy controls	The vaccine was safe in PWH; virus-specific antibody titers were significantly higher in controls than in PWH; PWH with higher CD4 counts had higher % of seropositivity; virus-neutralization titers against wild type and Delta variants were similar between PWH and controls	[121]
2022	Han et al.	Inactiva-ted SARS-CoV-2 vaccine; 2 doses	PWH and healthy controls without a prion infection with SARS-CoV-2	The vaccine was safe in PWH; neutralizing antibody responses to the D614G variant and delta variant were lower in PWH than controls; antibodies titers decreased but were maintained for 3 months; PWH with CD4 counts < 350 showed lower responses	[122]
2022	Spinelli et al.	2 doses; mRNA (BNT162b2 and mRNA-1273) vaccines	100 PWH and 1:1 matched HIV-healthy controls	PWH had lower surrogate virus neutralization and anti-RBD antibody titers; PWH also showed a trend towards lower IgG titers; the responses were particularly lower in PWH with lower CD4 counts; serological responses were higher in mRNA-1273 vaccinees	[123]
2022	Wu et al.	Inactivated virus vaccine; 2 doses	138 PWH and 35 HIV-healthy controls	Serocoversion rates were similar between PHW and controls 3 months post-vaccination; virus neutralization titers were lower in PWH; CD4 counts < 200 were associated with lower titers in PWH	[124]
2022	Hassold et al.	2 doses; mRNA vaccines or ChAdOx1	PWH with different CD4^+^ T cell counts	PWH with <500, in particular <200, CD4 counts had less S-specific antibody titers 1–3 months post-vaccination with BNT162b2, mRNA-1273 or ChAdOx1 nCoV-19	[119]
2022	Lamacchia et al.	4 doses; mRNA vaccines	8 PWH	S-specific CD4^+^ T cells transiently re-activated after 4th dose; and declined in two weeks; the 4th dose increased cross-reactive Omicron-specific MBC	[126]
2023	Polvere et al.	2 doses; mRNA vaccines	84 PWH and 79 healthy controls	S-specific antibodies lasted for six months; less response in PWH with CD4 counts < 350; comparable % of S-specific MBC in PWH and controls; PWH had higher % of DN and lower % of SMBC than controls	[125]
2023	Costiniuk et al.	3 doses of	294 PWH and 267 controls	S-specific IgG titers were similar at month 3 post 2nd dose and 1-month post 3rd dose in both cohorts; they declined earlier in some PWH after the second dose	[127]
2023	Lapointe et al.	3 doses of mRNA vaccine	99 PLWH and 152 healthy controls (some controls received 2 doses of ChAdOx1 and a third dose of mRNA vaccine	No evidence of faster decline in S-specific antibodies in PWH with undetectable viremia; 3rd dose increased Omicron-specific antibodies in PWH but they remained lower than in controls	[128]
2023	Touizer et al.	3 doses; mRNA vaccine (BNT162b2, mRNA-1273 or ChAdOx1)	110 PWH and 64 HIV-controls	Delayed development and reduced titers of virus-neutralizing antibodies linked to reduced numbers of S-specific MBC and cTFH in PWH; despite these cellular deficits, 3rd doses increases magnitude and breadth of antibody responses in PWH	[37]
2023	Cheung et al.	4 doses; mRNA vaccines	PWH with or without prior SARS-CoV-2 infection	4th dose enhances vaccine responses in PWH irrespective of prior SARS-CoV-2 experiences, and enhances WT and variant-specific antibodies	[129]
